# Accessibility of Cations to the Selectivity Filter of KcsA in the Inactivated State: An Equilibrium Binding Study

**DOI:** 10.3390/ijms20030689

**Published:** 2019-02-05

**Authors:** Ana Marcela Giudici, Maria Lourdes Renart, Clara Díaz-García, Andrés Morales, José Antonio Poveda, José Manuel González-Ros

**Affiliations:** 1 Instituto de Investigación, Desarrollo e Innovación en Biotecnología Sanitaria de Elche (IDiBE), and Instituto de Biología Molecular y Celular (IBMC), Universidad Miguel Hernández, Elche, E-03202 Alicante, Spain; marcela@umh.es (A.M.G.); lrenart@umh.es (M.L.R.); 2 CQFM-IN and IBB-Institute for Bioengineering and Bioscience, Instituto Superior Técnico, Universidade de Lisboa, 1049-001 Lisboa, Portugal; clara.dg93@gmail.com; 3 Departamento de Fisiología, Genética y Microbiología, Universidad de Alicante, E-03080 Alicante, Spain; Andres.Morales@ua.es

**Keywords:** potassium channels, C-type inactivation, selectivity filter conformation, protein thermal stability, fluorescence, ion-protein interactions

## Abstract

Cation binding under equilibrium conditions has been used as a tool to explore the accessibility of permeant and nonpermeant cations to the selectivity filter in three different inactivated models of the potassium channel KcsA. The results show that the stack of ion binding sites (S1 to S4) in the inactivated filter models remain accessible to cations as they are in the resting channel state. The inactivated state of the selectivity filter is therefore “resting-like” under such equilibrium conditions. Nonetheless, quantitative differences in the apparent K_D_’s of the binding processes reveal that the affinity for the binding of permeant cations to the inactivated channel models, mainly K^+^, decreases considerably with respect to the resting channel. This is likely to cause a loss of K^+^ from the inactivated filter and consequently, to promote nonconductive conformations. The most affected site by the affinity loss seems to be S4, which is interesting because S4 is the first site to accommodate K^+^ coming from the channel vestibule when K^+^ exits the cell. Moreover, binding of the nonpermeant species, Na^+^, is not substantially affected by inactivation, meaning that the inactivated channels are also less selective for permeant versus nonpermeant cations under equilibrium conditions.

## 1. Introduction

**Figure 1:**
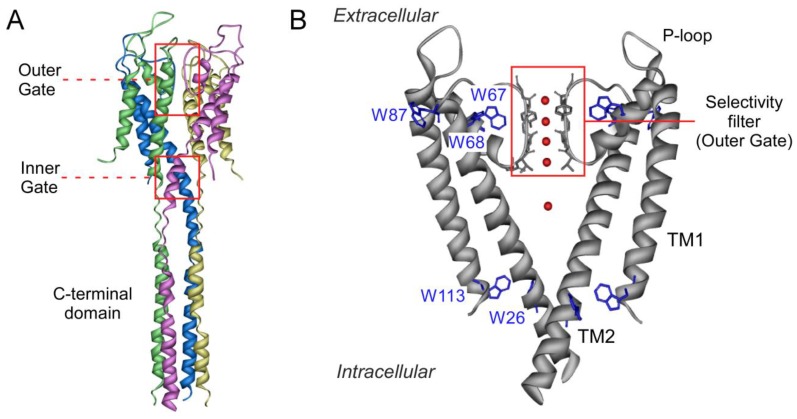
Schematic structure of the potassium channel KcsA. Panel **A** shows a side view of the full-length tetramer (PDB entry: 3EFF). Each monomer is highlighted in a different color. The location of the outer and inner gates, the latter in its closed conformation, is indicated by red squares. Panel **B** zooms in on the transmembrane portion of the channel (only two of the four identical subunits have been drawn for clarity) (PDB entry: 1K4C). Each monomer exhibits two transmembrane helices (TM1 and TM2), connected by the P-loop region and the selectivity filter (highlighted in red). Tryptophan residues are colored in blue and cations within the filter and in the channel vestibule appear as red spheres.

One of the most intriguing features of potassium channels is their high selectivity for the larger K^+^ ion versus the smaller Na^+^ ion, while maintaining a high ion flux [1]. MacKinnon’s [2] and Perozo’s [3] groups have contributed the most on this matter through the study of the structure of a potassium channel from *Streptomyces lividans*, KcsA. This channel is a homotetrameric membrane protein, where each monomer is conformed by two α-helical transmembrane segments (TM1 and TM2) and a C-terminal cytoplasmic end. The channel pore is found between TM1 and TM2, contributed by a short tilted helix and the selectivity filter [2], which also operates as a gate (outer gate). The four C-terminal ends arrange as a helical bundle with a conformation that is sensitive to pH, being another pore gate (inner gate) [3] (Figure 1). Importantly, the signature sequence of the selectivity filter of KcsA (TVGYG) is homologous to that of the eukaryotic K^+^ channels. The backbone carbonyls of these residues conform four K^+^ binding sites (sites S1–S4, from the extra to the intracellular side), as seen by crystallography [2,4], which can adopt different conformations at high or low K^+^ concentrations [4,5,6,7,8]. Nuclear magnetic resonance (NMR) studies have also reported such changes [9,10,11,12,13,14]. The conformation at low K^+^ concentration shows no ions at the center of the selectivity filter (sites S2 and S3), thus adopting a “collapsed” structure which impedes ion flow through it. K^+^ binds to S1 and S4 with an average occupancy of just one ion distributed between those two sites. However, at high K^+^ concentrations, a conformational change is induced whereby a second K^+^ enters the filter, with an average occupancy of two K^+^ ions per channel, either at the S1–S3 or the S2–S4 positions, thus enabling ion conduction [4,7]. The structure of KcsA in the presence of other permeating ions such as Tl^+^, Rb^+^ or Cs^+^ has also been studied through X-ray crystallography. In the case of the first ion, the occupation is identical to K^+^, while the other two ions are not able to bind the S2 site, although they still induce a conductive conformation with an average of two ions per channel [8]. The nonpermeant Na^+^, on the other hand, is not able to induce such a conformational change and shows an average occupancy of one ion per channel at the S1 and S4 sites [15].

In terms of functional activity, KcsA is described through a cycle including four different states. At neutral pH, the channel is in a closed/conductive resting state, whereby the cytoplasmic helical bundle (the inner gate) impedes ion flow, while the selectivity filter (the outer gate) displays a conductive form. At acidic pH, the inner gate opens, enabling the open/conductive state which allows ion flow, making KcsA a proton-activated channel [16,17,18]. However, this is not a stable state and the outer gate adopts a conformation reminiscent of C-type inactivation in eukaryotic K^+^ channels [19,20,21], which impedes ion flow within a few seconds [20,21,22] in the open/inactivated form. The cycle is completed when the pH returns to neutrality, which induces the closure of the inner gate and thus the transient closed/inactivated state, which rapidly evolves to the initial closed/conductive resting state [23]. This cycle reveals the concerted action of the two channel gates.

The elucidation of the molecular reasons for why the inactivated channel is nonconductive is of great interest to the mechanisms of ion conduction but is currently subject to controversy. Some authors relate the nonconductive, “collapsed” conformation of the selectivity filter seen in X-ray experiments at low K^+^ concentrations [4] (PDB entry: 1K4D) with the inactivated conformation [15], such that the access of K^+^ to the most internal S2 and S3 K^+^ binding sites would not be permitted [24]. In contrast, other authors have found only modest conformational changes in the G77 residue during inactivation [25] compared to the resting state, concluding that the selectivity filter remains “resting-like” upon inactivation, with all four K^+^ binding sites accessible to cations. Our goal in this paper is to contribute to this issue by studying ion binding under equilibrium conditions, as a tool to explore the access of cations to the ion binding sites within the selectivity filter of KcsA in the inactivated state, and to compare such accessibility with that which is exhibited by the channel in the resting state. Unfortunately, there is not a single consensus model for the inactivated state of KcsA and therefore, we used several “open/inactivated” channel models in our study. The first of such models is the wild-type (WT) KcsA at pH 4, which is the standard experimental system used to measure channel inactivation rates upon a pH jump from 7 to 4, which is known to maintain the opening the inner gate as long as the acidic condition is imposed [26,27]. Our second channel model is the deletion 1–125 KcsA that lacks the cytoplasmic 125 to 160 residues at the C-terminal helical bundle. Such deletion destabilizes the channel’s inner gate, and although it appears closed in the X-ray structure [2,4] (PDB entry: 1K4C), electron paramagnetic resonance data shows that in detergent solution at neutral pH it remains widely open [28]. Third and last, we use a full-length version of the so called H25R, R117Q, E120Q, R121Q, R122Q and H124Q sextuple mutant channel (OPEN) KcsA, in which a number of mutations, mostly at the C-terminal region (see Methods), result also in a permanently open inner gate, even at neutral pH [29].

To study ion binding to KcsA, we used a previously reported assay based on the intrinsic fluorescence from residues W67 and W68 of the channel, located at the short pore helix (Figure 1B), which are very sensitive to the selectivity filter conformation [2,6]. This fluorescent signal allows for the monitoring of the thermal denaturation of KcsA, which includes the dissociation of the tetrameric protein into its subunits and their partial unfolding [30,31,32], a process found to be dependent on the binding of ions to the selectivity filter [30]. The experimental observable in this assay, the apparent t_m_ (midpoint denaturation temperature in degrees Celsius) of the native to denatured thermal transition, is ideally suited to study cation binding [30,32], since it exhibits an extraordinarily wide range of cation-dependent changes. This allows the determination of apparent dissociation constants for the binding processes ranging from 10^−2^ to 10^−9^ M in the previously characterized closed state at pH 7.0 [30,33]. From these studies, it has been established that there is a single set of sites with low affinity (millimolar K_D_ values) for the nonpermeant cations such as Na^+^ or Li^+^, which have been associated to the crystallographic S1 and S4 sites. However, two different sets of sites have been found for the permeant K^+^, Rb^+^, Tl^+^, and even Cs^+^ ions, as their concentration increases. Previous studies by crystallography also evidence the ability of these cations to induce concentration-dependent transitions between nonconductive and conductive conformations of the selectivity filter. Based on this analogy, the high affinity set (micromolar K_D_ values) has been assigned also to the crystal S1 and S4 sites, thus securing displacement of competing nonpermeant cations. The second set of sites shows low affinity (millimolar K_D_ values), thus favoring cation dissociation and permeation, and is contributed by all S1–S4 crystallographic sites.

## 2. Results

### 2.1. Binding of TBA^+^

**Figure 2:**
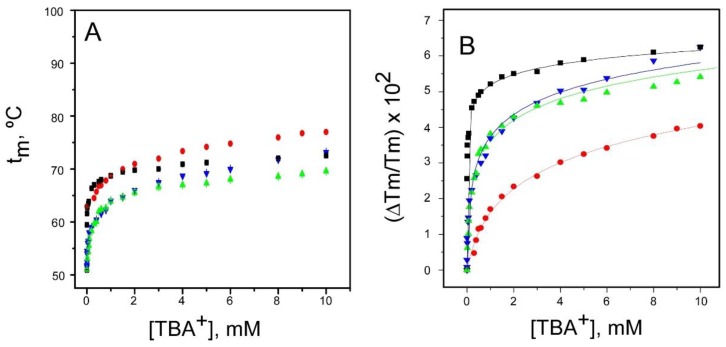
TBA^+^ binding to the resting and inactivated models of the KcsA channel. Thermal denaturation of DDM-solubilized KcsA was recorded in the presence of increasing concentrations of TBA^+^ by monitoring the temperature dependence of the protein intrinsic fluorescence. The midpoint temperature of the denaturation process (t_m_) at each TBA^+^ concentration was calculated from the thermal denaturation curves and plotted versus the TBA^+^ concentration. Panel **A** shows the results obtained from the resting KcsA channel (WT pH 7 (■) and from the three inactivated channel models (WT pH 4 (●); 1–125 (▲) and OPEN (▼) KcsA). All TBA^+^ titrations started with the channel proteins at a 1 µM concentration in either 20 mM Hepes (pH 7.0) or 10 mM succinic acid (pH 4.0) buffers, both containing 5 mM DDM and 1.5 mM NaCl. Here, as well as in all t_m_ vs. cation concentration plots in Figure 2, Figure 3, Figure 4, Figure 5, Figure 6, Figure 7 and Figure 8, the experimental results are the average t_m_ (in Celsius) ± SD at each cation concentration from three to four independent titrations. The term “independent” denotes that the samples were prepared from different protein stocks and the titrations were carried out on different days. Panel **B** illustrates the fitting of the average Tm (in kelvins) experimental data from panel **A** to the theoretical function given in Equation (1). The apparent dissociation constants estimated for the WT pH 7, WT pH 4, and the 1–125 and OPEN KcsA channels were 5 × (2.8–8.9) × 10^−9^, 3.5 × (2.9–4.3) × 10^−4^ M, 1.7 × (1.3–2.3) × 10^−5^ M and 1.7 × (1.2–2.5) × 10^−5^ M, respectively. In parentheses are the confidence intervals (CIs), using a percentage of confidence of 95%.

The inactivated channel models have an important feature in common regarding the binding of TBA^+^: A strong potassium channel blocker (Figure 2). In resting KcsA, TBA^+^ binds into the channel vestibule, blocking the entrance to the S4 ion binding site in the selectivity filter and interacting also with surrounding residues belonging to the selectivity filter (T75) and with the hydrophobic side-chains of I100 and F103 at the vestibular region of the M2 segments [34]. Crystal structures of KcsA with its cytoplasmic inner gate open at different degrees, as in the inactivated state, suggest a hinge-bending motion of the M2 segments in the closed to open channel transition. This bears on dragging apart the M2 segments within the transmembrane region, and thus, it is expected to broaden the TBA^+^ binding site. Other changes related to inner gate opening, such as a shortening of the helical pitch at the C-terminal of the pore helix and a change in the rotameric conformation of F103, have also been reported [3,24,35]. These structural alterations seem consistent with our observations that TBA^+^ binding is altered in the three inactivated channel models, with K_D_’s that are several orders of magnitude higher (lower affinities) than the nanomolar K_D_ observed in the binding to the resting channel state (inner gate closed) [33] (Figure 2). This suggests that under the experimental conditions used in these experiments (channel proteins at low concentration and in detergent solution), all three inactivated models are similarly open at their inner gates, meaning that the binding of TBA^+^ is similarly affected. Despite such similarity, it should be noted that the K_D_ value for the pH 4 KcsA model departs somewhat from the very similar K_D_ values estimated for the 1–125 and OPEN KcsA models, both at pH 7. In any case, as a corollary from the above, it appears that the large changes detected in TBA^+^ binding to the inactivated models could be useful as a simple diagnostic tool for inner gate opening, and likely for channel inactivation.

### 2.2. Binding of Na^+^

**Figure 3:**
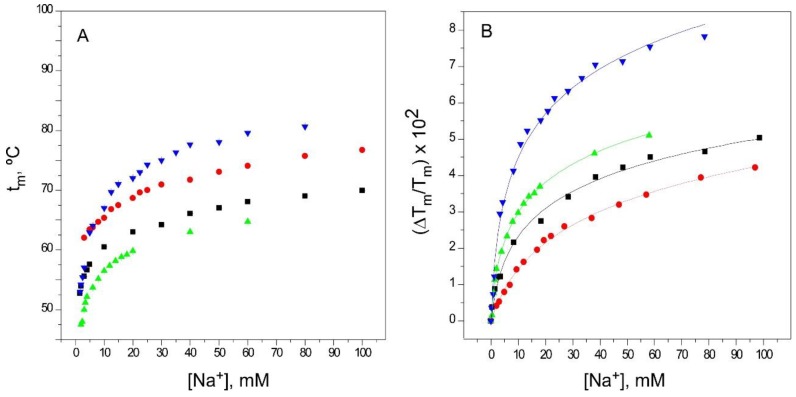
Binding of Na^+^ to KcsA. Panel **A** illustrates Na^+^ binding to KcsA channels, monitored through the Na^+^ concentration dependence of the t_m_ of thermal denaturation. The results are the average (*n* = 3) t_m_ (in Celsius) ± SD. Symbols and colors are the same as in Figure 2. Panel **B** shows the fitting of the experimental data from Panel A to Equation (1) (see Methods). The apparent K_D_ values for the above binding events are given in Table 1.

Na^+^ is the physiologically-relevant, nonpermeant species to K^+^ channels. Equilibrium binding of Na^+^ to the nonconductive KcsA can be described by the occupation of a single set of sites contributed by the S1 and S4 binding sites in the selectivity filter [15,30] (PDB entry: 2ITC). Figure 3A shows that increasing the Na^+^ concentration from 1.5 mM to 100 mM or higher, causes a different degree of thermal stabilization in the different protein models. Nonetheless, as in the resting KcsA model, all Na^+^ binding curves can be fitted to a single binding event (Figure 3B) with estimated K_D_’s in the millimolar range (Table 1). Again, the 1–125 and OPEN models, both at pH 7, behave very similarly to each other, with K_D_ values practically identical to that of the resting KcsA model, while the pH 4 model departs somewhat from such behavior. Overall, however, this indicates that under equilibrium conditions, the accessibility of Na^+^ to the S1 and S4 sites at the selectivity filter in all three inactivated channel models is comparable to that seen in the resting KcsA.

### 2.3. Binding of Ba^2+^

**Figure 4:**
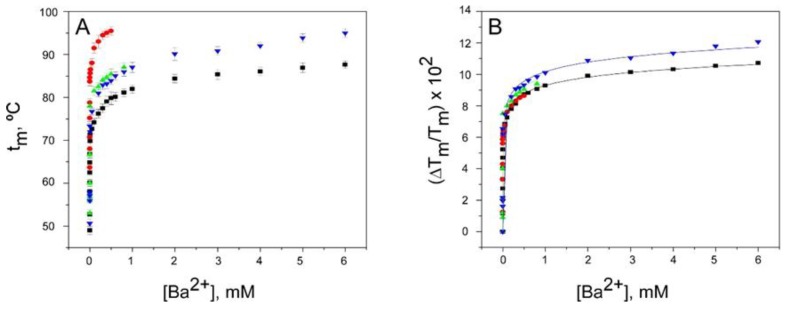
Binding of Ba^2+^ to KcsA. Panel **A** illustrates Ba^2+^ binding to KcsA channels, monitored through the Ba^2+^ concentration dependence of the t_m_ of thermal denaturation. The results are the average (*n* = 3) t_m_ (in Celsius) ± SD. The symbols and colors are the same as in Figure 2. Panel **B** shows the fitting of the experimental data from Panel A to Equation (1) (see Methods). The apparent K_D_ values for the above binding events are given in Table 1.

The divalent Ba^2+^ is a strong K^+^ channel blocker which binds with very high affinity to the S2 and S4 sites of the selectivity filter [15]. Figure 4 shows that in the three inactivated KcsA models, equilibrium binding of Ba^2+^ can be described by a single binding event characterized by nanomolar K_D_’s (Table 1), almost identical to that reported previously in the resting state [32]. This suggests that the accessibility of the divalent cation to the selectivity filter of the different channels is very similar both in the resting and in the inactivated states.

### 2.4. Binding of K^+^

**Figure 5:**
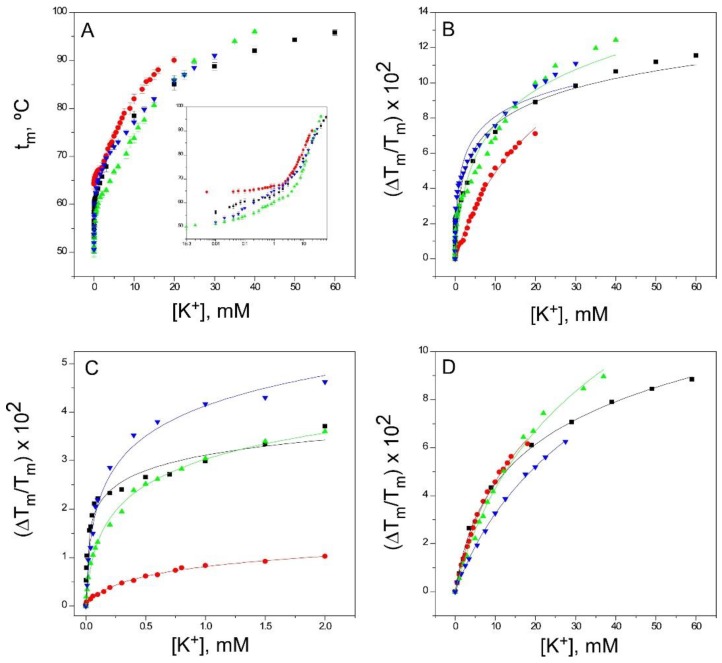
Binding of K^+^ to KcsA. Panel **A** shows K^+^ titrations covering the widest possible range of concentrations (i.e., those increasing the t_m_ to near the thermostat limit of our circulating water bath) conducted on KcsA samples containing 1.5 mM Na^+^ as the starting point. The results are the average (*n* = 3) t_m_ (in Celsius) ± SD. The symbols and colors are the same as in Figure 2. Inset to panel A are semi-log plots of the binding processes to illustrate in a simple manner the presence of two different sets of K^+^ binding sites. Indeed, fitting the data from panel A to Equation (1) fails when taking into account the whole titration curve (i.e., assuming a single set of binding sites) (Panel **B**), but it suffices when the low (Panel **C**) and the high (Panel **D**) K^+^ concentration ranges are analyzed separately, suggesting that at least two different sets of K^+^ binding sites are present in the KcsA samples. The analysis of the binding data to more than one set of binding sites and the determination of the apparent dissociation constants for the individual binding events, as in Figure 5, Figure 6 and Figure 7, have been described in detail in the Supplementary Information to Reference [33]. The apparent K_D_ values estimated for the above binding events are given in Table 1.

K^+^ is the physiologically-relevant permeant species to K^+^ channels. Previous studies of equilibrium binding to the resting state of KcsA [30] have shown that K^+^ binds to two different sets of sites as its concentration increases, which is consistent with the crystallographic evidence on the ability of K^+^ to induce a concentration-dependent transition between nonconductive and conductive conformations of the selectivity filter. The first set of such sites, assigned to the crystal S1 and S4 sites [4], shows a high affinity for K^+^ (K_D_ in the micromolar range), thus securing the displacement of competing nonpermeant cations. The second set of sites results from the contribution of all S1 through S4 crystallographic sites in the selectivity filter, is available only to the permeant cation when the filter is in the conductive conformation and shows low affinity (K_D_ in the millimolar range), thus favoring K^+^ dissociation and permeation [7,30]. Figure 5 shows that K^+^ binding to the three inactivated channel models also exhibited biphasic binding curves, comparable to that seen in the resting state, suggesting that, under our experimental equilibrium conditions, the accessibility of K^+^ to all sites within the selectivity filter remains similar upon inactivation. Nonetheless, there is a clear trend in the three inactivated models in which the K_D_ values for such binding processes are increased with respect to the resting state (Table 1). Particularly, the K_D_ for the first, higher affinity binding event, increases by an order of magnitude in the 1–125 and OPEN channels and even further in the pH 4 model. Such binding events are likely the basis of cation selectivity and conduction by the channel, and therefore, the results suggest that the inactivated channels are less selective for the permeant cation than the channel in the resting state.

**Figure 6:**
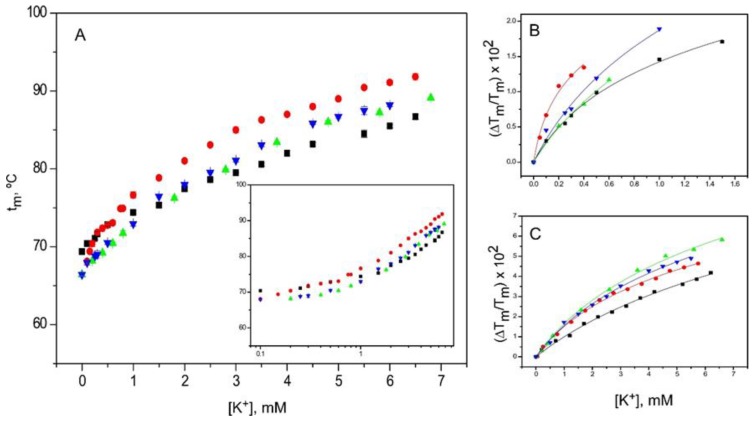
Effects of TBA^+^ blockade on the binding of K^+^ to KcsA. Panel **A** shows titration experiments conducted on KcsA samples as in Figure 5, but in the continuous presence of 10 mM TBA^+^. The results are the average (*n* = 3) t_m_ (in Celsius) ± SD. The symbols and colors are the same as in Figure 2. Inset to panel A is a semi-log plot that still reveals the presence of two sets of K^+^ binding sites, even under TBA^+^ blockade. Panels **B** and **C** show the fitting of the experimental data from the low and high K^+^ concentration ranges, respectively, to Equation (1). The apparent K_D_ values derived from such fittings are included in Table 1.

K^+^ binding experiments were also conducted in the presence of a saturating concentration of TBA^+^ (Figure 6). There was a blockade of the S4 site by TBA^+^ in these experiments, however, it may have been compromised by the lower affinity for TBA^+^ shown by the three inactivated channel models compared to the resting state, which may have resulted in only a partial blockade or in the displacement of the bound TBA^+^ by the increasing concentration of K^+^. Nonetheless, in addition to the lower extent of thermal stabilization of the protein channels observed upon K^+^ binding, the K_D_ values obtained in the presence of TBA^+^ differed from those obtained in its absence (Table 1). This suggests that the TBA^+^ blockade of K^+^ binding was operative under our experimental conditions. Additionally, the results show that, as in the resting state, the 1–125 and OPEN inactivated channels exhibited K_D_ values for the first, higher affinity binding event, which are more than an order of magnitude higher (lower affinity) than those in the absence of TBA^+^, while the pH 4 model seems almost unaffected by the presence of TBA^+^ (Table 1). In the resting state, the first binding event at low K^+^ concentration takes place to a nonconductive selectivity filter, and therefore, the results obtained under the TBA^+^ blockade should reflect primarily the binding of K^+^ to the S1 site. Conversely, at a higher K^+^ concentration (i.e., binding to the second set of sites), binding of the cation to the 1–125 and OPEN inactivated models under TBA^+^ blockade showed an apparent increase in affinity, which was not observed in the resting state (Table 1).

### 2.5. Binding of Rb^+^ and Cs^+^

Both Rb^+^ and Cs^+^ are nonphysiological, permeant species to K^+^ channels, whose permeation is greatly hindered by the fact that none of them can occupy the S2 site in the selectivity filter [8]. This poses a serious energetic barrier to ion flow, increasing the residence time of these cations within the filter and lowering the rates of inactivation [27,32].

**Figure 7:**
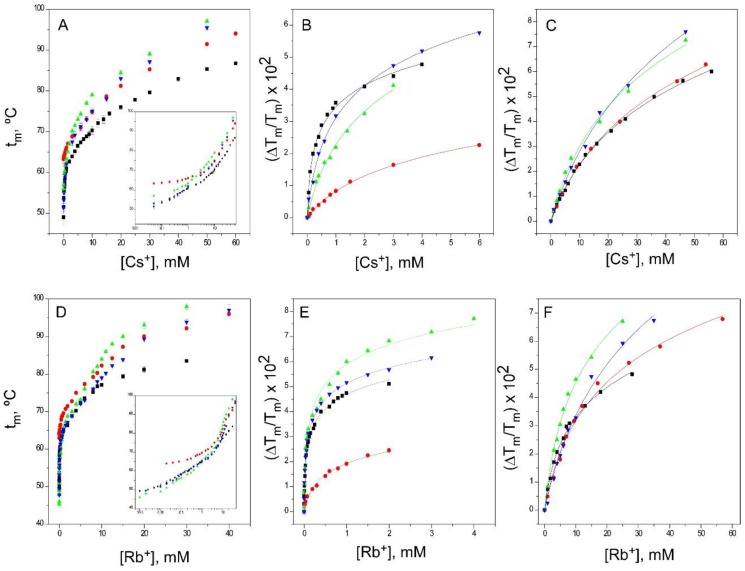
Binding of Cs^+^ and Rb^+^ to KcsA. The changes in the apparent t_m_ with the concentration of Cs^+^ and Rb^+^ (Panels **A** and **D**, respectively) allows the detection of two sets of thermodynamically different binding sites, as illustrated in the insets to Panels A and D. The results are the average (*n* = 3) t_m_ (in Celsius) ± SD. Panels **B** and **E** show the fitting of the experimental data to Equation (1) for the first sets of binding sites for the two cations (low concentration range), whereas Panels **C** and **F** show the fits for the second set of binding sites (high concentration range). The symbols and colors are the same as in Figure 2. The apparent K_D_ values calculated for these experiments are included in Table 1.

As in the resting state, binding of these two cations to the inactivated models also exhibited biphasic binding curves (Figure 7), comparable to that seen for K^+^ binding, suggesting that the sequential, concentration-dependent binding processes that relate first to selectivity and finally to the permeation of these cations were also operative in the inactivated states. Thus, these results indicate that the accessibility at equilibrium of both cations to the S1, S3 and S4 sites within the selectivity filter remains similar upon inactivation. Again, the pH 4 model departs somewhat from the behavior observed in the 1–125 and OPEN channels.

As different from the observations with the physiologically-relevant K^+^, binding of Rb^+^ does not clearly detect a lower affinity in its binding to the inactivated channel models, except for the first event in the pH 4 model. In contrast, such a decrease in affinity is observed in the binding of Cs^+^, although it is restricted to the first binding event only (Table 1).

### 2.6. Effects of an Acidic pH on the Binding of Cations to the Inactivated Channel Models

**Figure 8:**
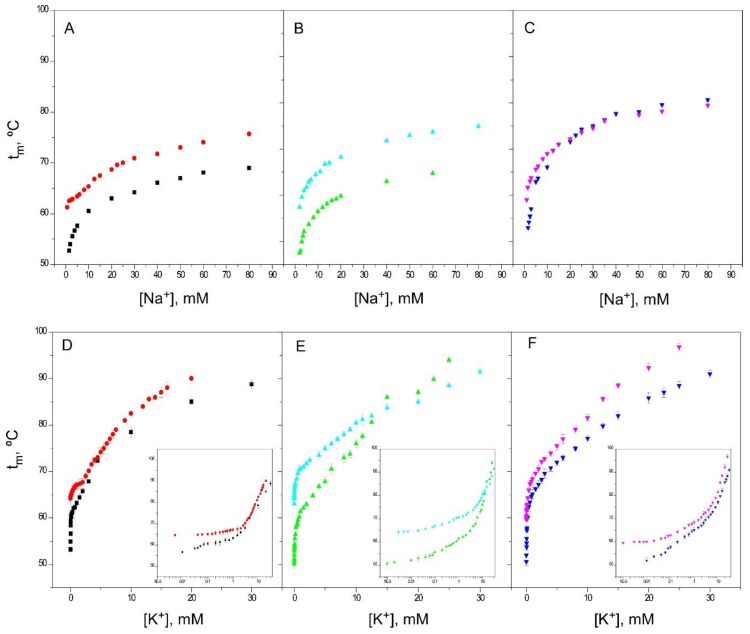
Effect of pH 4 on the thermal stability of the different KcsA models. Panels **A**, **B** and **C** compare the Na^+^ binding profiles, while Panels **D**, **E** and **F** show the K^+^ binding profiles (and the corresponding semi-log plots as insets) of the same KcsA samples prepared at pH 7 and pH 4. The results are the average (*n* = 3) t_m_ (in Celsius) ± SD. Symbols and colors are WT KcsA at pH 4 (●) and pH 7 (■); 1–125 KcsA at pH 4 (▲) and pH 7 (▲) and OPEN KcsA at pH 4 (▼) and pH 7 (▼).

In the previous paragraphs, the binding of different ions to the inactivated models (1–125 and OPEN KcsA, both at pH 7, and wild-type KcsA at pH 4) have been compared to that of the resting channel state (wild-type KcsA at pH 7). In such comparisons, the pH 4 model often differed quantitatively from the two other inactivated models at neutral pH, and therefore, additional experiments were carried out with the 1–125 and OPEN KcsA models at pH 4, in an attempt to evaluate the influence of an acidic pH “per se” on the binding of ions to these model channels. Figure 8 illustrates such an influence for the binding of both Na^+^ and K^+^. In essence, it was observed that the acidic pH condition increases the thermal stability of the proteins studied throughout most of the titration curves. Our thermal denaturation assay relies precisely on the ability of the cations to increase the thermal stability of the protein in a cation-specific, concentration-dependent manner. Therefore, the results obtained at an acidic pH, where an additional contribution to the thermal stability occurs, should be interpreted with caution. These additional pH effects could partly explain the somewhat diverging results seen in the pH 4 model compared to the other two inactivated models used in the study.

## 3. Discussion

**Figure 9:**
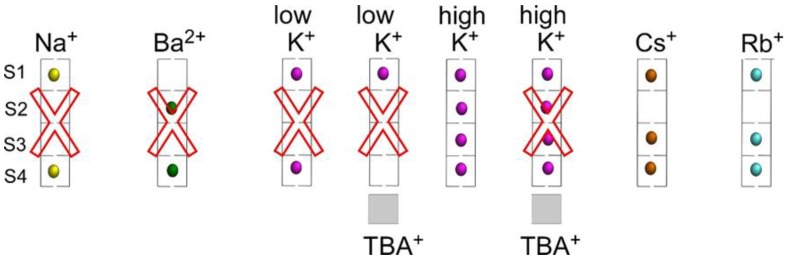
Idealized potential occupation of the KcsA selectivity filter by Na^+^, Ba^2+^, K^+^, Cs^+^ and Rb^+^, in the presence or absence of TBA^+^ bound to the channel’s vestibule. Red crosses indicate nonconductive conformations for the selectivity filter under those experimental conditions. The PDB entries used for these models are: 2ITC (Na^+^), 2ITD (Ba^2+^), 1K4D (low K^+^), 2HVJ (low K^+^, TBA^+^), 1K4C (high K^+^), 2HVK (high K^+^, TBA^+^), 1R3L (Cs^+^) and 1R3I (Rb^+^).

The accessibility of different cations to the stack of K^+^ binding sites in the selectivity filter of the KcsA channel has been previously documented by X-ray crystallography and by a variety of other techniques [2,4,9,11,14,15,28,36]. Regardless of the use of different KcsA proteins (full length, mutations or partial deletions of the channel), most of this information has been obtained under equilibrium conditions, in detergent solution and at neutral pH, at which the channel is in the resting state when in the presence of adequate cations, i.e., with a closed inner gate and a conductive selectivity filter. Figure 9 summarizes such potential occupations of the selectivity filter by cations in an idealized manner. The nonpermeant Na^+^ binds with low affinity to the S1 and S4 sites of a nonconductive filter. Likewise, the strong potassium channel blocker Ba^2+^ binds with an extremely high affinity to the S2 and S4 sites. On the other hand, the permeant K^+^ at low concentration binds with fairly high affinity to the S1 and S4 sites of a nonconductive, “collapsed” filter. Blocking the access to the S4 site by TBA^+^ at these low concentrations of K^+^ allows for K^+^ binding to the S1 site only. Finally, at higher K^+^ concentrations, there is a cation-induced change in the conformation of the selectivity filter to a conductive state, by which K^+^ gain access with roughly equal probability to all S1 to S4 sites through a permeation-allowing binding event, characterized now by a lower affinity. Likewise, Cs^+^ or Rb^+^ are both permeant species which behave similarly to K^+^, with the exception that at high concentrations these two cations bind to the S1, S3 and S4 sites only, but cannot access the S2 site (Figure 9).

**Figure 10:**
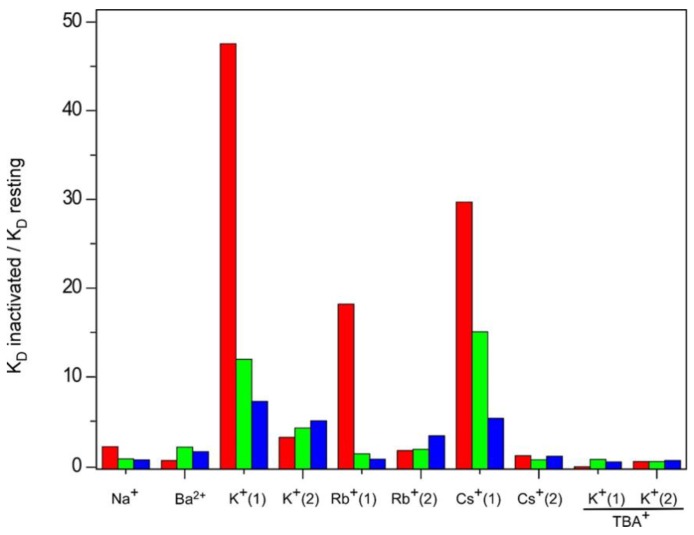
Relative affinities for nonpermeant and permeant cations presented by the three KcsA inactivated model channels, with respect to the resting state. The bar graph shows the ratio between the mean apparent K_D_’s of the inactivated and resting states given in Table 1. Colors represent: KcsA at pH 4 (■), 1–125 KcsA at pH 7 (■) and OPEN KcsA at pH 7 (■).

In this report we have used a thermal denaturation assay to study the equilibrium binding of all the above cations to three different models of KcsA in the inactivated state. Our goal was to explore possible changes in the accessibility of the cations to the stack of K^+^ binding sites in the selectivity filter as a consequence of channel inactivation. Two major hypotheses on this matter are currently entertained. The first hypothesis postulates that the inactivated state of the selectivity filter corresponds to a “collapsed” conformation, similar to that seen in presence of low K^+^ concentration [15] (PDB entry: 1K4D), in which access to the most internal S2 and S3 sites is not permitted [24]. On the contrary, the second hypothesis claims that the filter remains in a “resting-like” conformation upon inactivation, with all the K^+^ binding sites similarly accessible to cations [28]. In any case, either hypothesis should be able to explain why the inactivated channel is nonconductive. Our results show that qualitatively, all the binding sites within the inactivated selectivity filter, probed either individually or collectively though the binding of the different cations, remain as accessible as in the resting state. Therefore, we have no evidence to support a “collapsed” filter conformation with inaccessible sites in the inactivated state and must conclude that the inactivated filter is “resting-like” in terms of cation accessibility under equilibrium conditions. In spite of such a similarity, the quantitative comparison between the equilibrium binding constants derived from the resting channel state and from the three inactivated models points out differences which refer mainly to a decreased affinity in the binding of permeant cations to the inactivated channels (Figure 10). Such a decreased affinity seems more clearly observed for K^+^ binding, especially in the first binding event. This implies that the initial binding of K^+^ to the S1 and S4 sites, which occurs at a low cation concentration and is proposed to be the basis for ion selectivity [30], is altered in the inactivated versus the resting state. In the case of the pH 4 inactivated channel, such an affinity is nearly 50-fold lower than that in the resting state. As indicated above, the results from the pH 4 model should be taken with caution, because of the additional effect of pH on the thermal stabilization of the channel protein. Nonetheless, the 1–125 and OPEN channel models also detect a decrease in the affinity for binding of permeant species, and therefore, we conclude that this is a common qualitative feature in all three inactivated models. 

Experiments of K^+^ binding under blockade of the S4 site by TBA^+^ could serve to evaluate whether the S1, S4 or both of these sites are involved in the decreased affinity of the first K^+^ binding event exhibited by the inactivated channels. Again, these experiments should take into account the much lower affinity of TBA^+^ for binding to the inactivated state versus the resting state of the channel, which might not completely guarantee an efficient TBA^+^ blockade of K^+^ binding. Furthermore, it is possible that the TBA^+^ blockade could additionally differ in terms of efficacy among the three inactivated models. In spite of such potential limitations, the results seem to point out two main findings: First, similarly to the resting state [33], the inactivated filter remains asymmetric, since the affinity for K^+^ binding to the extracellular S1 site is lower, relative to the intracellular S4 site. Second, comparing the resting versus the inactivated states, the changes in the K_D_’s for K^+^ binding under TBA^+^ blockade were not as large as those seen in its absence (Figure 10). Therefore, it could be concluded that K^+^ binding to the S1 site was less affected by inactivation than that in the S4 site. Thus, the S4 site appears to be mainly responsible for the observed loss in affinity during the first event of K^+^ binding. This observation seems to be in agreement with the model proposed by Valiyaveetil et al., in which inactivation was suggested to affect mainly the S3 and S4 sites of the selectivity filter [37].

Thus, in overall terms, the most noticeable change between the resting and the inactivated state of KcsA from our binding experiments is a drop in the affinity for permeant ions, especially notorious for K^+^, which is precisely the ion that promotes deeper and faster channel inactivation. As indicated throughout the manuscript, similarly to X-ray crystallography or NMR, our experiments were done under equilibrium conditions. Still, it is tempting to compare our results with those from electrophysiology, although they are under kinetic control and have a voltage imposed to define the direction of the flow of ions. In the latter, inactivation has long been associated to a loss of K^+^ at the selectivity filter in potassium channels [38,39], which explains why C-type inactivation is favored at low K^+^ concentrations in eukaryotic potassium channels [4,15,19,40], while a higher concentration of the ion prevents it [27,40,41,42,43]. These observations led to the “foot in the door” or the “ion depletion of the pore” hypothesis, which basically proposed that the presence of ions inside the selectivity filter is fundamental to stabilize it in the conductive conformation [44,45,46]. The drop in the affinity we detected in our equilibrium binding experiments would increase the probability of K^+^ loss from the filter, then hampering ion conduction. As for the collapsed conformation found for the inactivated state in the crystallization experiments [24], it is conceivable that such a conformation was reached temporarily, favored by the affinity drop, where a kinetic intermediate could perhaps become stabilized under the crystallization conditions. In fact, the very essence of the selectivity filter is to be dynamic, subject to continuous sojourns to different conformations, which explains the discrete closures in the conductive state and the different gating modes of KcsA [47,48]. 

Another consequence from the drop in potassium affinity related to the partial loss of selectivity for K^+^ versus Na^+^. Since sodium affinity seems to not be significantly altered in the inactivated state, the K_D_ (Na^+^)/(K_D_ (K^+^) ratio diminishes. This parameter is related to ion selectivity only at equilibrium [30,32,33,49], but could perhaps be used to explain the reported loss of selectivity in several potassium channels, observed electrophysiologically when in the inactivated state [50,51,52]. In extreme cases, such as that in the case of the inactivated M96V-KcsA channel model, the loss of selectivity is so severe that in addition to K^+^, the usually nonpermeant Na^+^ becomes a permeant species in this mutant channel [53].

In summary, use of cation binding as a tool to explore the accessibility at equilibrium of permeant and nonpermeant cations to different inactivated channel models shows that the stack of ion binding sites in the inactivated filter qualitatively remain accessible to cations, as in the resting channel state. The inactivated selectivity filter is therefore “resting-like” under such equilibrium conditions. Nonetheless, quantitative differences in the K_D_’s of the binding processes reveal the affinity for binding of permeant cations, mainly K^+^, to the inactivated channel models is decreased with respect to the resting channel state. This is likely to cause a loss of K^+^ from the inactivated filter, and consequently, to promote nonconductive conformations. S4 seems the most affected site by the affinity loss, which is interesting because S4 is the first site to accommodate K^+^ coming from the channel vestibule under physiological conditions of K^+^ exit from the cell.

## 4. Materials and Methods

### 4.1. Protein Expression and Purification

H25R, R117Q, E120Q, R121Q, R122Q and H124Q mutations (“OPEN” mutant) were introduced to the *kcsA* gene by PCR site-directed mutagenesis (Genewiz, South Plainfield, NJ, USA). The mutations were confirmed by dideoxynucleotide sequencing. The N-terminal hexahistidine tagged wild-type (WT) and OPEN KcsA channels were expressed in *Escherichia coli* M15 (pRep4) cells and purified in good quantity by immobilized metal affinity chromatography on Ni^2+^-Sepharose resin (GE Healthcare, Madrid, Spain), as previously described [54]. The protein stock at 50 µM was then dialyzed in a 20 mM HEPES buffer (pH 7.0) or a 10 mM succinic acid buffer (pH 4.0), both containing 5 mM DDM (Calbiochem) and 75 mM NaCl. The C-terminal deleted 1–125 KcsA mutant was obtained from the full-length WT KcsA by treatment with α-chymotrypsin-agarose beads (from bovine pancreas, Sigma, Madrid, Spain) at 37 °C for 90 min with constant agitation at 200 rpm. The hydrolysis was stopped by centrifugation at 18,000× *g* at 4 °C and subsequently the supernatant was collected. Protein concentration was routinely determined from the absorbance at 280 nm, using a molar extinction coefficient of 34,950 M^−1^ cm^−1^ for the WT and OPEN channels and 33,460 M^−1^ cm^−1^ for the 1–125 mutant [55]. Tetramer integrity at pH 7.0 and 4.0 was routinely checked in the three model channels by SDS-PAGE in 12% polyacrylamide gels [56]. All the referred salts and buffers were purchased from Sigma-Aldrich.

For functional characterization, the WT, OPEN and 1–125 KcsA channels were reconstituted into asolectin giant liposomes and their ion channel activity was recorded by patch-clamp methods [57]. A residual KcsA activity, with a very low opening probability, was observed at pH 4 in all cases, indicative of inactivated channels [47]. As expected, the OPEN KcsA model shows such a residual activity also at pH 7, confirming that its inner gate remains open regardless of the pH used in the experiments.

### 4.2. Fluorescence Monitoring of Cation Binding

Thermal denaturation of detergent DDM-solubilized proteins in the presence of increasing amounts of the different cations was performed in a Varian Cary Eclipse spectrofluorometer (Agilent), by monitoring the temperature dependence of the protein intrinsic fluorescence emission at 340 nm after excitation at 280 nm. The temperature up-scan rate was 0.6 °C/min [30,31,53]. Batteries of samples were prepared from the dilution of the protein stock to 1 µM KcsA final protein concentration in a 20 mM Hepes (pH 7.0) buffer or a 10 mM succinic acid (pH 4.0) buffer, both containing 5mM DDM and 1.5 mM NaCl. The final concentration of 1.5 mM NaCl is the minimal cation concentration at which the WT KcsA at pH 7.0 still remains tetrameric and shows a sigmoidal thermal denaturation. The three inactivated channel models at 1.5 mM NaCl also show a tetrameric structure in SDS-PAGE and show a sigmoidal thermal denaturation, and therefore, this Na^+^ concentration was adopted as the initial condition in all titrations to facilitate the comparison of all the different new samples with those reported previously for the WT KcsA at pH 7.0 [30,32]. Aliquots from KCl, RbCl, CsCl, NaCl, BaCl_2_ or tetrabutylammonium chloride (TBA Cl) stock solutions were subsequently added into these samples to provide the desired amount of each tested salt. In the case of the TBA^+^ blockade experiments, all samples contained a constant concentration of 10 mM TBA^+^ and increasing amounts of K^+^.

The midpoint temperature of the thermally-induced protein denaturation process in the different samples (T_m_) at the different cation concentrations was calculated from the thermal denaturation curves by fitting a two-state unfolding model to the data, assuming a linear dependence of the pre- and post-transition baselines on temperature [58]. The concentration-dependent increase in T_m_ was used to estimate the dissociation constant, K_D_, of the protein-ligand (cation) complex using the following equation:

(1) $$\frac{\Delta T_{m}}{T_{m}}=\frac{T_{m}-{\left(T_{m}\right)}_{0}}{T_{m}}=\frac{R\cdot {\left(T_{m}\right)}_{0}}{\Delta H_{0}}\cdot \ln\left[1+\frac{\left[L\right]}{K_{D}}\right]$$

where T_m_ and (T_m_)_0_ refer to the denaturation temperature (in kelvins) for the protein in the presence and absence of a given concentration of added ligand (L), respectively. R is the gas constant and ΔH_0_ is the enthalpy change upon protein denaturation in the absence of added ligand [30,53].

## Figures and Tables

**Table 1:** Apparent dissociation constants (K_D_’s) for the binding of different cations to the resting and inactivated state models of the KcsA channel. Mean values given here come from the experiments reported in Figure 3, Figure 4, Figure 5, Figure 6 and Figure 7. Since the estimated K_D_ values are derived from a logarithmic function (Equation (1)), we use the 95% confidence intervals of these values for statistical comparisons, instead of giving mean ± SD values, since only parametric analysis is appropriate on the logarithmic scale for such data. ^a^ Significant differences with respect to the resting WT, pH 7 group (*p* < 0.05). ^b^ Significant differences with respect to the same sample without 10 mM TBA (*p* < 0.05).

Tested Cations	Sets of Binding Sites Detected	Resting Channel		Inactivated Channel Models					
		WT KcsA, pH 7		WT KcsA, pH 4		1–125 KcsA		OPEN KcsA	
		K_D_ (M)	95% CI	K_D_ (M)	95% CI	K_D_ (M)	95% CI	K_D_ (M)	95% CI
Na^+^	1	3.3 × 10^−3^	(2.5–4.3) × 10^−3^	7.7 × 10^−3^	(6.7–8.9) × 10^−3 a^	3.1 × 10^−3^	(2.9–3.3) × 10^−3^	2.7 × 10^−3^	(2.5–2.9) × 10^−3^
Ba^2+^	1	4.0 × 10^−9^	(2.7–6.0) × 10^−9^	3.0 × 10^−9^	(1.3–6.9) × 10^−9^	9.0 × 10^−9^	(3.0–27) × 10^−9^	7.0 × 10^−9^	(2.9–17) × 10^−9^
K^+^	2	1.9 × 10^−6^	(1.2–2.9) × 10^−6^	9.0 × 10^−5^	(7.4–11) × 10^−5 a^	2.3 × 10^−5^	(2.0–2.7) × 10^−5 a^	1.4 × 10^−5^	(1.1–1.8) × 10^−5 a^
		2.5 × 10^−3^	(1.8–3.5) × 10^−3^	8.4 × 10^−3^	(7.5–9.4) × 10^−3 a^	1.1 × 10^−2^	(9.3–13) × 10^−3 a^	1.3 × 10^−2^	(1.1–1.5) × 10^−2 a^
K^+^, 10 mM TBA	2	8.0 × 10^−4^	(6.4–9.9) × 10^−4 b^	6.0 × 10^−5^	(3.6–9.9) × 10^−5 a^	7.0 × 10^−4^	(4.5–11) × 10^−4 b^	4.9 × 10^−4^	(4.5–5.3) × 10^−4 a,b^
		3.2 × 10^−3^	(2.4–4.2) × 10^−3^	2.1 × 10^−3^	(1.8–2.5) × 10^−3 b^	2.1 × 10^−3^	(1.8–2.5) × 10^−3 b^	2.4 × 10^−3^	(2.1–2.8) × 10^−3^
Rb^+^	2	4.0 × 10^−6^	(3.5–4.6) × 10^−6^	7.3 × 10^−5^	(7.1–7.5) × 10^−5 a^	6.0 × 10^−6^	(4.5–8.0) × 10^−6^	3.7 × 10^−6^	(3.0–4.5) × 10^−6^
		3.4 × 10^−3^	(2.8–4.2) × 10^−3^	7.0 × 10^−3^	(6.1–8.0) × 10^−3 a^	7.5 × 10^−3^	(6.9–8.1) × 10^−3 a^	7.4 × 10^−3^	(6.7–8.2) × 10^−3 a^
Cs^+^	2	3.1 × 10^−5^	(2.5–3.9) × 10^−5^	9.2 × 10^−4^	(8.8–9.6) × 10^−4 a^	4.7 × 10^−4^	(3.5–6.3) × 10^−4 a^	1.7 × 10^−4^	(1.4–2.1) × 10^−4 a^
		1.3 × 10^−2^	(1.1–1.5) × 10^−2^	1.7 × 10^−2^	(1.5–1.9) × 10^−2 a^	1.1 × 10^−2^	(9.3–13) × 10^−3^	1.6 × 10^−2^	(1.3–2.0) × 10^−2^

## Abbreviations

KcsA	potassium channel from *Streptomyces lividans*
1–125 KcsA	KcsA deletion that lacks the cytoplasmic 125 to 136 residues
OPEN	H25R, R117Q, E120Q, R121Q, R122Q and H124Q sextuple mutant channel
DDM	dodecyl β-d-maltoside
TM	transmembrane segment
SDS–PAGE	sodium dodecyl sulfate–polyacrylamide gel electrophoresis
t_m_	midpoint denaturation temperature (in degrees Celsius)
T_m_	midpoint denaturation temperature (in kelvins)
ΔH_0_	enthalpy change upon protein unfolding in the absence of ligand
K_D_	dissociation constant
TBA^+^	tetrabutylammonium cation
